# Social Determinates of Health Among Older Adults Living With HIV/AIDS

**DOI:** 10.1093/geroni/igab046.1634

**Published:** 2021-12-17

**Authors:** Erin Robinson, Tonya Taylor, Charles Emlet

## Abstract

In the United States (U.S.), people aged 55 years and older make up 36% of people living with HIV/AIDS (PLWHA). Nearly 20% of new HIV infections occur among people aged 50+. While medical breakthroughs in HIV treatment have allowed PLWHA to live longer, healthier lives, structural conditions still exist that affect health outcomes of older adults living with HIV/AIDS (OALWHA). These conditions continue to disproportionately burden OALWHA, particularly older adults of color. Therefore, a greater understanding of the social determinates of health (SDH) is essential to continue making progress in HIV treatment, maintenance, and prevention. The U.S. Centers for Disease Control and Prevention (CDC) has highlighted several SDH among OALWHA, including: poverty, education, income, employment status, health insurance coverage, and housing. This symposium will highlight emerging research that examines several of these indicators among OALWHA. Using a variety of research methodologies, the five abstracts included in this symposium aim to address: 1) psychosocial risk factors of quality of life; 2) life instability and mental health; 3) institutional barriers and facilitators of successful aging; 4) determinants of engaging in advance care planning; and 5) a needs assessment of OALWHA, with particular emphasis on SDH. Results from this research identify several priority areas (such as housing instability, mental health, food insecurity, and isolation) for healthcare leaders to consider in targeting future policy, programming, and funding. Future initiatives are essential to help continue the progress in HIV/AIDS treatment and prevention, including addressing SDH among the aging population living with HIV/AIDS.

